# Posttraumatic stress and symptom improvement in Norwegian tourists exposed to the 2004 tsunami – a longitudinal study

**DOI:** 10.1186/1471-244X-13-232

**Published:** 2013-09-24

**Authors:** Ajmal Hussain, Lars Weisæth, Trond Heir

**Affiliations:** 1 Norwegian Centre for Violence and Traumatic Stress Studies, University of Oslo, Postboks 181, Nydalen, Oslo 0409, Norway; 2 Faculty of Medicine, University of Oslo, Oslo, Norway

**Keywords:** 2004 tsunami, Longitudinal study, Posttraumatic stress, Norwegian tourists, Personality, Witness exposure, Social support, Treatment

## Abstract

**Background:**

Mental health consequences of disasters are frequently studied. However, few studies have investigated symptom improvement in victims after natural disasters. This study aimed to identify predictors of 6 months post-disaster stress symptoms and to study 6 months and 24 months course of symptoms among Norwegian tourists who experienced the 2004 tsunami.

**Methods:**

Norwegian tourists (≥18 years) who experienced the 2004 tsunami (n = 2468) were invited to return a postal questionnaire at two points of time. The first data set was collected at 6 months (T1, n = 899) and the second data set at 24 months post-disaster (T2, n = 1180). The population studied consisted of those who responded at both assessments (n = 674). Impact of Event Scale Revised (IES-R) was used to measure posttraumatic stress symptoms. IES–R score ≥33 (caseness) was used to identify various symptom trajectories from T1 to T2. Multiple linear regression was used to determine predictors of posttraumatic stress at T1 and to identify variables associated with symptom improvement from T1 to T2.

**Results:**

The majority was identified as non-case at both assessments (57.7%), while 20.8% of the respondents were identified as case at both assessments. Symptoms at T1 were positively related to female gender, older age, unemployment, being chased or caught by the waves, witnessing death or suffering, loss of loved ones, experiencing intense fear during the disaster, low conscientiousness, neuroticism and low levels of social support. The IES-R sum score declined from 24.6 (SD = 18.5) at T1 to 22.9 (SD = 18.3) at T2, p < 0.001. Emotional stability and high IES-R scores at T1 were positively related to symptom improvement, while received social support was not. Being referred to a mental health specialist was negatively related to symptom improvement.

**Conclusions:**

A significant minority (20-30%) among Norwegian tourists developed enduring posttraumatic stress symptoms in the aftermath of the 2004 tsunami. Tsunami exposure, peritraumatic fear, neuroticism and low levels of social support were the strongest predictors of posttraumatic stress at 6 months post-disaster. Decrease in posttraumatic stress was related to emotional stability and higher symptom levels at T1. Being referred to a mental health specialist did not facilitate symptom improvement.

## Background

Mental health consequences of disasters and particular natural disasters have been frequently studied [1]. The relation between intensity of traumatic event exposure and risk of posttraumatic stress symptoms is well established [2-4]. In addition, a number of pretrauma vulnerability factors (e.g female gender, psychiatric history, and specific personality traits), peritraumatic reactions (e.g. life threat, fear, dissociation) and post-trauma factors (e.g. lack of social support, and other life stressors) are identified as predictors of posttraumatic stress [2,5,6]. The inverse relationship between posttraumatic stress and social support is one of the most consistent relationships observed in trauma research [2,5]. Social support is one of the most frequent factors which may prevent PTSD onset [7], or help trauma survivors to cope with severe posttraumatic stress symptoms [8].

Although the course of posttraumatic stress symptoms may vary according to type of trauma, most disaster studies show that symptoms are prominent in close proximity to the traumatic event and most often decline within the first year [4,9,10]. However, a significant number of disaster victims develop chronic symptoms that last for many years [4,10-14]. Guidelines for treatment of posttraumatic stress is well established [15]. However, treatment-effects obtained within research setting, i.e expert psychotherapists in specialized centers, may not generalize to real-world effectiveness [16].

Some common concerns have been raised by several authors when reviewing and evaluating the disaster research [1,17]. For instance, due to the nature of disastrous events, it is common to use a convenience sample that may limit generalization. Studies of whole populations are rarely conducted. The majority of the studies conducted are cross-sectional in design, which makes it difficult to draw conclusions about cause-effect relationships. In case of studies with longitudinal design, most have their last assessment within 1 year post-disaster. Besides, it is pointed out that the effect of specialized treatment for PTSD is often neglected when assessing outcome (posttraumatic stress).

Natural disasters generate massive destruction, often destroying the entire infrastructure of communities, forcing survivors to cope with loss of homes or livelihood as well as lack of support and treatment facilities and uncertainty about the future [4,18]. Thus, it is hard to differentiate between symptoms caused by direct exposure to the disaster (primary stressors) and symptoms caused by the consequences of disaster damage (secondary stressors). Besides, people living in areas prone to natural disasters, must face the constant threat of new disasters. A common observation is that the mental health impact of natural disasters is generally lighter compared to other types of disasters [4]. However, this may partially be explained by tendency to include persons less directly exposed in studies of natural disasters [3,19]. The mental health effect following a tsunami has not been studied prior to the tsunami in 2004.

All of the Norwegian tourists visiting in South East Asia at the time of the tsunami were repatriated to stable home communities within short time [20]. This unforeseen situation resulted in a unique possibility to study mental health affects following a fatal natural disaster among a whole population of tourists who escaped secondary disaster stressors. The present study was part of “*The Tsunami research program*” conducted by The Norwegian Centre for Violence and Traumatic Stress Studies.^a^ This study aimed to investigate predictors of posttraumatic stress and to identify factors that influence the long-term course of symptoms over time in a tourist population. We were particularly interested in studying the primary effect of disaster exposure and the effect on symptom improvement in accord with social support, personality and being referred to a mental health specialist.

## Methods

### Procedure and participants

Norwegian tourists who had resided in a country affected by the 2004 tsunami were repatriated in the days following the tsunami and registered by the police upon their arrival in Norway. Permission from the Norwegian Data Inspectorate (Datatilsynet) and the Regional Committee for Medical Research Ethics (Regional Etisk Komité for medisinsk forskning) was obtained to make this information available for our study.

**Figure 1:**
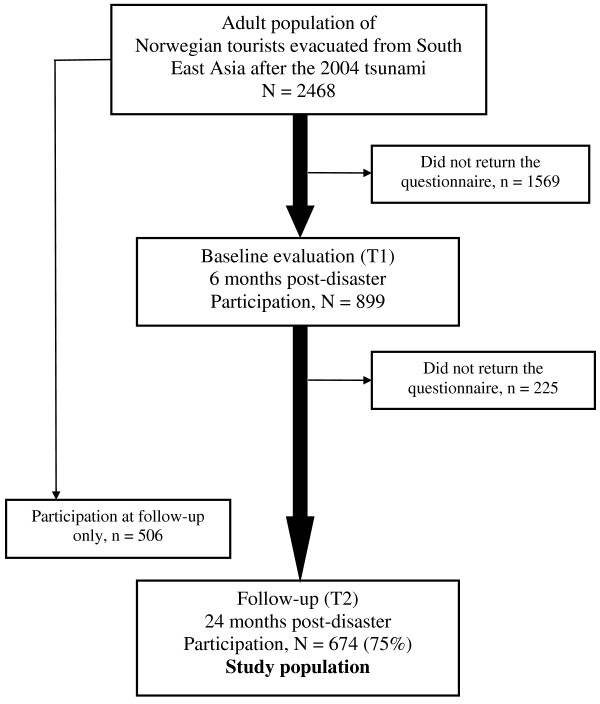
**Flow-chart of participants in the study.** A postal questionnaire was sent to all Norwegians aged 18 or above who were repatriated to Norway after the tsunami (n = 2468) at two time points; 6 months postdisaster (T1) and two years postdisaster (T2). Response rate at T1 was 36% (n = 899) and 48% (n = 1180) at T2, whereas 674 individuals responded at both times and constituted the study population (27% of the total population). 225 persons were lost to follow up resulting in 75% response rate at T2 according to T1-population, whereas 506 persons responded at T2 only.

A postal questionnaire was sent to all the registered individuals aged ≥18 years (n = 2468) at two points of time: 6 months post-disaster (T1) and 24 months post-disaster (T2). A unique tracking number was attached to the questionnaires to prevent them from getting mixed up. Our population consisted of those who responded at both assessments (n = 674, see Figure 1). We sent a reminder for returning the first questionnaire few weeks after the initial distribution. At T2 the questionnaire was shortened and we sent two reminders following the initial distribution.

An information letter was sent along with the postal questionnaire. The letter contained information regarding the study and about methods for handling and storing data confidentially and stated that participation was voluntary. The persons were asked to fill in and return the questionnaire if they agreed with the terms and conditions mentioned in the information letter.

The mean age in the study sample was 43.0 years (SD = 13.0), 46.7% were men, 58.9% had ≥13 years of education, 73.4% were employed (before tsunami) and 71.8% were married or cohabitating (before tsunami) (Table 1).

**Table 1:** Background characteristics of the study population and survey question frequency data (N = 674)

		
Age at time of tsunami, years (mean, range)	43.0, 18-81	
	n	%
Gender		
Male	315	46.7
Female	359	53.3
Married or cohabiting prior to the tsunami		
Yes	477	71.8
No	187	28.2
Married or cohabiting at T1		
Yes	452	70.3
No	191	27.7
Education		
< 13 years	276	41.1
≥ 13 years	396	58.9
Employed prior to the tsunami		
Yes	495	73.4
No	179	26.6
Employed at T1		
Yes	467	69.3
No	207	30.7
Pre-tsunami mental health problems		
No	505	75.7
Yes	162	24.7
Chased or caught by the waves		
No	424	64.3
Yes	235	35.7
Witnessed abandoned children, death or suffering of others		
No	249	37.9
Yes	408	62.1
Loss of family members or close friends		
No	622	92.7
Yes	49	7.3
Experienced intense fear, helplessness, or horror		
No	191	30.7
Yes	431	69.3
Referred to a mental health specialist		
No	611	90.7
Yes	63	9.3
Experienced ≥2 adverse life events last 12 months at T2		
No	438	65.6
Yes	230	43.4

#### Study sample and non-responders

The non-responders at T1 were similar in age but consisted of a higher proportion of men [21]. The attrition rate from T1 to T2 was 25%. Compared to the study sample the drop-outs were younger and had been exposed to danger to a lesser degree. There were no other statistical differences with regard to socio-demographic variables, exposure, peritraumatic reactions, social support, and posttraumatic stress. Among those who participated at T2 only there were more men compared to the study sample. T2 participants exclusively were also younger, had been exposed to danger to a lesser degree and had lower levels of posttraumatic stress.

Generally, the response rate was much higher in subgroups that had resided in the most severely affected areas, and it was correspondingly lower among those who had been in locations that were less severely affected [21,22].

We investigated lack of participation with follow-up telephone interviews in a random sample of non-responders at T2 [23]. Non-participants reported lower levels of disaster exposure and lower levels of posttraumatic stress symptoms. The most frequently reported reasons for not participating were lack of interest or time (39.2%) and not being directly affected by the disaster (32.2%).

### Measures

The questionnaires included a variety of commonly used variables used in post disaster mental health research covering pre-, peri-, and post-disaster aspects [1,17]: previous mental health, personality traits, disaster exposure, loss of loved ones, peritraumatic fear, social support, seeking professional help and exposure to other adverse life events. A more detailed discussion regarding the selection of the variables is given in a previous publication [21].

#### Pre-disaster aspects

The following demographic and background variables were measured at T1: gender, age, educational level (≥13 years indicating high educational level), employment status (pre-tsunami and at T1), and marital status (married or cohabitating prior to the tsunami and at T1).

Pre-tsunami mental health problems were measured by asking whether respondents had ever contacted a general practitioner or a mental health professional due to mental health problems prior to the tsunami disaster.

#### Personal characteristics of participants

The 44-item Big Five Inventory was used to measure five personality dimensions [24,25] at T2. Higher scores are associated with Extroversion (I), Agreeableness (II), Conscientiousness (III), Neuroticism (IV), and Openness (V). Neuroticism is the only factor associated with non-desirable behaviours and therefore sometimes reversed and called Emotional Stability. In this paper the term neuroticism is being used to describe and discuss level of posttraumatic stress. When discussing improvement, the term emotional stability is being used. Scores for each of the subscales were used as continuous measure in the analysis.

#### Tsunami exposure and peritraumatic fear

At the 6-month assessment, the questionnaire included a broad spectrum of tsunami experiences [21]. Potential traumatic exposure were explored according to whether a participant had been caught, touched or chased by the waves (exposure to danger); witnessed death and suffering of others (witnessing exposure); or had a close relative or friend who died. The exposure variables were not mutually exclusive.

Participants were asked whether their immediate response were characterized by fear, helplessness or horror. The responses were measured on a five-point scale: 0, not at all; 1, little; 2, moderate; 3, intense; 4, extreme. A score of 3 or 4 was considered as a positive response to Criterion A2 for PTSD in DSM–IV [26] and is being used as a dichotomized measure of peritraumatic fear.

#### Post-disaster aspects

##### Social support

The Crisis Support Scale (CSS) [27] consists of seven items and was used to measure received social support in the 6 months after the tsunami. CSS includes two dimensions of support, i.e. total social support (first six items) and satisfaction with support (last item). We used the scale to measure total social support which concern the availability of others, contact with other people in similar situation, confiding in others, emotional and practical support, and negative response. Each item is rated on a 7-point Likert scale ranging from 1 (never) to 7 (always). The negative response is reverse scored. Items were summed up to yield a total score of social support score ranging from 6 to 42. In general, a higher score indicates higher levels of social support. Scale-scores were used as a continuous *variable i*n the analysis.

#### Referral to a mental health specialist

At the 6 months follow-up participants were asked whether they had been referred to a mental health specialist following the tsunami. The scores were coded as “no” (0) or “yes” (1).

#### Adverse life events

Participants completed a 12-item life-event inventory at the 24-month assessment [28]. The number of adverse life events that a participant experienced during the previous 12 months was recorded: Serious illness or injury to oneself or close relative, bereavement (family or close friend), end of relationship, problem with close friend or relative, difficulty finding a job, sacked from job, financial crisis, problems with police/law and theft/loss. Positive responses were arranged in two groups, experienced 0–1 event or ≥2 events.

#### Posttraumatic stress

In both assessments, the 22-item Impact of Event Scale–Revised (IES–R) [29] was included to examine the presence and intensity of post-traumatic stress symptoms (intrusion, avoidance, and hyperarousal) during the previous week. The participants responded to each item on a five-point Likert scale (0–4) regarding their experience with the tsunami. The IES–R total symptom scores (range 0–88) were used as semi-continuous measures of symptom severity. In this study, the Cronbach alpha for the total scale was 0.96 at both assessments. Means for each of the subscales (intrusion, avoidance/numbing and hyperarousal) were used to compare changes in symptom intensity from T1 to T2. IES-R was suitable for our study. The questionnaire has proved appropriate for use in non clinical settings when measuring general level of posttraumatic stress is of interest rather than the PTSD diagnosis.

We used a cut-off score of ≥33 to identify caseness according to IES-R, which is a cut-off recommended to provide the best diagnostic accuracy to identify persons with high levels of posttraumatic stress [30]. Caseness was used to identify various symptom trajectories from T1 to T2. In an additional post hoc evaluation of symptom improvement according to whether participants were referred to a mental health specialist or not, the analysis was restricted to participants with initial IES–R score ≥33.

### Statistics

Responders and non-responders were compared by using independent *t*-test for continuous variables and Chi-square test for categorical variables. Paired samples *t*-test was used to examine changes in symptom scores from T1 to T2.

We used hierarchical multiple linear regression analysis to determine the adjusted effects of potential predictors of IES-R score at T1. Assumptions for linear regression were tested and found satisfactory [31]. This analysis was performed in four steps where each step contained a group of variables arranged in a theoretical and practical manner: Step 1 included socio-demographic variables such as age, gender, education, employment, marital status, and pre-tsunami mental health problems. Step 2 constituted the additional inclusion of exposure and peritraumatic fear. Step 3 included Big Five personality traits and in step 4 social support was included. To identify variables associated with symptom improvement the difference between IES-R scores at T2 and T1 were entered as dependent variable in a similar hierarchical multiple linear regression model. We wished to control for IES-R score at T1 (step 2). Thus, variables regarding exposure and peritraumatic fear were left out because they did not make any practical contribution to the model. In addition, these variables gave little clinical meaning as high levels of exposure and peritraumatic fear were associated with improvement. In the last step additional information regarding consulting a mental health specialist and experiencing additional adverse life events was included.

All analyses were conducted using the software Statistical Package for the Social Sciences (SPSS, version 16.0). All the categorical variables were dichotomized before running the analysis (Table 1). P-values <0.05 were considered significant.

## Results

The reported pre-tsunami lifetime prevalence of contact with a general practitioner or a mental health professional for mental health concerns was 24.3%. The majority of respondents experienced disaster exposure; 62.1% witnessed death or suffering of others, 35.7% were chased or caught by the waves and 7.3% reported that a close family member or friend perished in the tsunami. A vast majority (69.3%) also reported experiencing intense fear, helplessness, or horror during the disaster (peritraumatic fear). At T1, 63 persons (9.3%) reported that they had been referred to a mental health specialist following the tsunami. The two most frequent reported adverse life events were bereavement of distant relative or close friend (22.0%) and problem with close friend or relative (20.8%). Of the respondents at T2, 34.4% had experienced two or more adverse life event during last 12 months (Table 1).

### Predictors of posttraumatic stress 6 months post-disaster

Table 2 shows predictors of post traumatic stress at 6 months post-disaster (T1). In the final four step model female gender, increasing age, unemployment, disaster exposure i.e. witnessing death or suffering, being chased or caught by the waves and loss of loved ones, peritraumatic fear, low conscientiousness, neuroticism and low levels of social support were associated with posttraumatic stress. Peritraumatic fear, witnessing death or suffering and neuroticism were the strongest predictors of posttraumatic stress at T1 in the final model. The total explained variance was 43% (R = 0.65).

**Table 2:** **Multiple linear regression - prediction of posttraumatic stress (IES-R**^**1**^**) at 6 months postdisaster, N = 674**

	**Step 1 Background**			**Step 2 + exposure and peritraumatic reactions**			**Step 3 + personality factors**			**Step 4 + post-disaster factors**		
	**β**^**2**^	**t**	**p-value**	**β**^**2**^	**t**	**p-value**	**β**^**2**^	**t**	**p-value**	**β**^**2**^	**t**	**p-value**^**3**^
Female	0.15	3.71	<0.001	0.09	2.59	0.010	0.08	2.13	0.034	0.10	2.63	**0.009**
Age at time of tsunami (years)	0.01	0.27	0.786	0.08	2.18	0.030	0.10	2.81	0.005	0.09	2.65	**0.008**
High educational level	−0.13	−3.38	0.001	−0.09	−2.58	0.010	−0.04	−1.16	0.245	−0.04	−0.98	0.327
Unemployed (pre-disaster)	0.11	2.76	0.006	0.12	3.37	0.001	0.11	3.15	0.002	0.08	2.40	**0.017**
Living alone (pre-disaster)	−0.06	−1.44	0.150	0.01	0.20	0.84	−0.02	−0.44	0.657	−0.02	−0.65	0.515
Pre-tsunami mental health problems	0.06	1.60	0.111	0.05	1.28	0.195	−0.05	−1.34	0.179	−0.04	−1.17	0.241
Chased or caught by the waves				0.19	5.23	<0.001	0.18	5.19	<0.001	0.18	5.00	**<0.001**
Witnessed abandoned children, death or suffering of others				0.23	6.34	<0.001	0.24	6.82	<0.001	0.23	6.57	**<0.001**
Loss of family members or close friends				0.11	3.04	0.003	0.09	2.66	0.008	0.09	2.68	**0.008**
Experienced intense fear, helplessness, or horror (peritraumatic fear)				0.28	7.67	<0.001	0.25	7.04	<0.001	0.24	6.83	**<0.001**
Extraversion (I)							−0.05	−1.30	0.194	−0.02	−0.59	0.558
Agreeableness (II)							0.04	1.04	0.298	0.05	1.37	0.171
Conscientiousness (III)							−0.08	−2.02	0.044	−0.09	−2.14	**0.032**
Emotional stability (IV)							−0.25	−5.74	<0.001	−0.24	−5.45	**<0.001**
Openness (V)							<0.01	0.05	0.959	<0.01	0.10	0.922
Social support										−0.14	−4.12	**<0.001**

^1^Impact of Event Scale Revised.

^2^Standardized partial regression coefficient, indicate the effect of each independent variable on the dependent variable controlled for all the other independent variables in the regression model.

^3^P-values <0.05 are highlighted with bold script in the final step.

### Changes in posttraumatic stress symptoms

**Figure 2:**
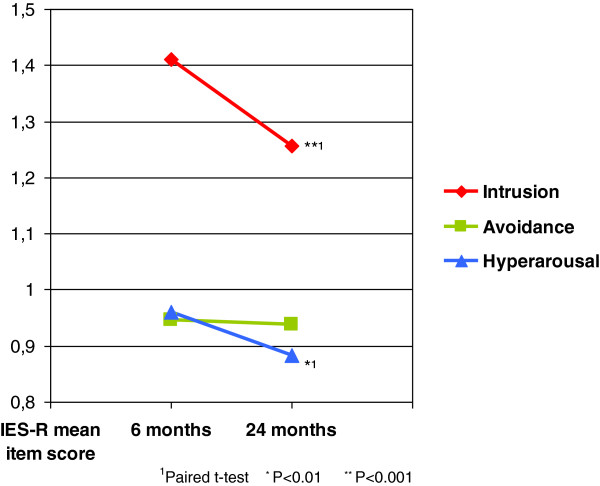
**Change in IES-R mean item score from 6 months postdisaster to 24 months postdisaster (T2) split into three clusters.** Mean item score of Impact of Event Scale Revised (IES-R), 0–4, is used to show change in the three clusters of the scale from 6 months to 2 years after the tsunami. Statistical test showed a significant reduction in Intrusion and Hyperarousal subscale scores but not in Avoidance subscale-score.

The total IES-R score declined from 24.6 (SD = 18.5) at T1 to 22.7 (SD = 18.3) at T2, *p* <0.001. At cluster level, we found a significant reduction in both intrusion and hyperarousal symptoms with the greatest reduction in the intrusion score (Figure 2). There was no significant reduction in avoidance score. According to caseness based on IES-R total score ≥33, the majority were identified as non-case at both assessments (57.7%). About 11 percent of the respondents went from being case at T1 to non-case at T2. On the other hand 7.4% went from being non-case at T1 to case at T2. A considerable number of respondents were identified as case at both assessments (20.8%) with relatively high IES-R total scores, 49.3 (SD = 12.2) at T1 and 48.4 (SD = 12.1) at T2, p = 0.352.

### Symptom improvement

Regression analyses showed that high IES-R score at T1 and emotional stability were both positively related to symptom improvement from 6 to 24 months (Table 3). Having been referred to a mental health specialist was negatively related to symptom improvement. We further explored the effect of having been referred to a mental health specialist by analysing symptom change in a subpopulation with high level of posttraumatic stress at T1 (IES-R cut-off ≥33). Significant symptom improvements were found whether participants were referred to a mental health specialist or not (Table 4). In this subpopulation there was no significant difference in symptom improvement between those who had been referred to a mental health specialist and those who had not (*p* = 0.269).

**Table 3:** **Multiple linear regression - predictors of symptom improvement (IES-R**^**1 **^**score) from 6 months postdisaster to 24 months postdisaster, N = 674**

	**Step 1 Background**			**Step 2 + IES-R (T1)**			**Step 3 + personality factors**			**Step 4 + post-disaster factors**		
	**β**^**2**^	**t**	**p-value**	**β**^**2**^	**t**	**p-value**	**β**^**2**^	**t**	**p-value**	**β**^**2**^	**t**	**p-value**^**3**^
Female	0.04	0.96	0.338	−0.02	−0.37	0.711	−0.01	−0.09	0.925	−0.01	−0.15	0.884
Age at time of tsunami (years)	−0.01	−0.29	0.772	<−0.01	−0.13	0.901	−0.05	−1.21	0.228	−0.04	−1.04	0.297
High educational level	0.06	1.48	0.140	0.10	2.69	0.007	0.06	1.40	0.161	0.08	1.96	0.051
Unemployed (at T1)	0.05	1.09	0.275	−0.04	−1.05	0.292	−0.03	−0.88	0.379	−0.03	−0.75	0.454
Living alone (at T1)	−0.01	−0.26	0.796	−0.01	−0.23	0.819	−0.01	−3.79	0.705	0.02	0.42	0.678
Pre-tsunami mental health problems	−0.03	−0.81	0.421	−0.05	−1.23	0.218	0.05	1.25	0.213	0.08	1.94	0.053
IES-R score at T1				0.39	9.83	<0.001	0.51	12.5	<0.001	0.54	12.6	**<0.001**
Extraversion (BFI-I)							−0.04	−0.81	0.420	−0.03	−0.60	0.550
Agreeableness (BFI-II)							−0.01	−0.12	0.907	−0.01	−0.24	0.811
Conscientiousness (BFI-III)							0.05	1.10	0.274	0.03	0.75	0.454
Emotional stability (BFI-IV)							0.33	6.43	<0.001	0.32	6.06	**<0.001**
Openness (BFI-V)							0.03	0.67	0.505	0.02	0.44	0.657
Social support										0.01	0.28	0.780
Referred to a mental health specialist										−0.12	−2.17	**0.002**
Experienced ≥2 adverse life events last 12 months at T2										−0.08	−1.93	0.054

^1^Impact of Event Scale Revised.

^2^Standardized partial regression coefficient, indicate the effect of each independent variable on the dependent variable controlled for all the other independent variables in the regression model.

^3^P-values <0.05 are highlighted with bold script in the final step.

**Table 4:** **Comparison of changes in IES-R**^**1 **^**scores from 6 months postdisaster (T1) to 24 months postdisaster (T2) among respondents with high level of posttraumatic stress at T1 (IES-R ≥33)**

	**IES-R (T1)**	**IES-R (T2)**	**Improvement in IES-R**		
	**Mean (95% CI)**	**Mean (95% CI)**	**Mean (95% CI)**	**Statistical test**^**2**^	**p-value**
Referred to a mental health specialist (n = 42)	50.4 (46.7-54.9)	45.3 (39.9-50.6)	5.10 (0.80-9.39)	2.40	0.021
Not referred (n = 171)	45.8 (44.0-47.5)	37.9 (35.4-40.3)	7.90 (5.56-10.2)	6.92	<0.001

^1^Impact of Event Scale Revised.

^2^Paired samples *t*-test.

Table 4 shows mean of total IES-R score among respondents with high level of posttraumatic stress at T1 (IES-R ≥33, n = 213) stratified by whether they were referred to a mental health specialist or not. The decline in IES-R score in both groups is tested for statistical significance.

## Discussion

The majority of Norwegian tourists in our study had relatively low levels of posttraumatic stress symptoms 6 months post-disaster (T1). High symptom level at T1 was related to female sex, older age, unemployment, exposure, peritraumatic fear, personality traits such as neuroticism and low conscientiousness, and low levels of social support.

### Effect of exposure

In agreement with many studies of natural disasters, we found that disaster exposure and peritraumatic reactions were among the strongest predictors of posttraumatic stress [3,32-35]. The results of our study also are comparable to studies of Thai and Sri Lankan tsunami survivors [36-38]. We found that witnessing abandoned children, seriously injured persons or dead bodies were more strongly related to posttraumatic stress than exposure to water or loss of loved ones. The pathologizing effect of horror is well documented in studies of children witnessing domestic violence [39] and soldiers witnessing atrocities [40,41], but is often given less importance when assessing exposure in disaster survivors [42]. Witnessing horror was also a key variable in predicting general psychological distress in a previous study of Norwegian tourists [21]. Our findings indicate that witnessing horror can be just as, or even more, traumatizing than the exposure to danger. Exposure to grotesque sights should therefore be taken into account when conducting surveys of disaster survivors [43]. It is still unclear whether the impact of witness exposure is more severe as a part of survival than persons who are only exposed to such grotesque sights in the aftermath. Some studies do suggest that witness exposure without danger to life constitutes a lesser risk for enduring posttraumatic stress symptoms [21,44].

### Symptom trajectories

We found a slight but significant decrease in the IES-R sum score from 6 months to 24 months post-disaster. The decrease in posttraumatic stress was more modest than generally expected after natural disasters [4,33]. This expectation was particularly well-founded as we studied a population who escaped secondary stressors and returned to stable home communities after a short period of time [19,20,45]. It is likely that among many tourists the duration of significant posttraumatic stress reactions were less than 6 months, thus resulting in little additional decrease in symptom level from 6 months to 24 months post-disaster. In addition, many of the included persons were not directly exposed to the dangers of the tsunami and may have experienced little or no symptoms in the aftermath. However, the finding also indicates that many who were markedly exposed and experienced personal life threat or suffered loss developed enduring posttraumatic stress symptoms.

At first look our results may presume that Norwegian tourists experienced little variation in level of posttraumatic stress from T1 to T2. However, based on status of being case (IES-R total score ≥33), we could identify various symptom trajectories. This is in agreement with other follow-up studies of posttraumatic stress symptoms [37,46] and with the understanding that persons with enduring symptoms often experience a fluctuating course rather than a stable and consistent level of posttraumatic stress [47-49].

### Effect of referral on symptom improvement

We found that consulting mental health specialists did not facilitate symptom improvement, which was somewhat surprising as we expected that treatment would alter the course in favor of symptom reduction. However, similar findings were reported in a follow-up study after the NYC terror attacks [50]. The National comorbidity data showed that in more than one third of the affected individuals the symptoms persisted for many years even when treatment was given [13]. It is argued that symptoms may be maintained and triggered by day-today adverse life experience. In addition, it is likely that secondary trauma stressors, such as loss of home or livelihood, play an important role in development of enduring posttraumatic stress following natural disasters [51]. However, the majority of our population was protected from secondary adversities as they were able to return to unaffected home communities.

One may argue that the regression analysis (Table 3) solely represent the fact that persons referred to a mental health specialist are those with highest levels of posttraumatic stress. Further investigation of this finding did show a slightly higher symptom level at T1 among persons referred to a specialist (Table 4, analyses restricted to persons with IES-R cut-off ≥33). However, in this subpopulation (IES-R cut-off ≥33), there was no significant difference in symptom improvement between persons referred and not referred to a mental health specialist. These findings do not support a negative relation between referral and symptom improvement, but they do indicate lack of efficacy of treatment given in specialized mental health care. Nevertheless, these findings must be interpreted with caution as our study was not designed as an effect-study.

It is important to bear in mind that we asked the participants whether they had been referred to a mental health specialist instead of asking directly about receiving treatment in the specialist healthcare. The question was chosen to include people who were referred to a specialist, but had not yet received treatment at the time of the first survey (T1). One may question whether the patients in this study actually received proper treatment or were just referred to treatment. However, a considerable part of those referred to treatment was satisfied by the support received from psychiatric services, which indicates that they actually received some kind of treatment [52]. Furthermore, if some persons declined the treatment offered as a result of symptom improvement, this would not change our findings regarding lack of treatment efficacy.

We did not measure details about the treatment received in specialist mental health care, but other studies of Norwegian tsunami survivors imply that common treatment received in specialised setting was supportive psychotherapy with or without the use of psychotropic drugs [53]. Our results may question the effectiveness of psychiatric treatment of enduring posttraumatic stress symptoms. It also raises concern about a possible negative effect of psychiatric treatment on symptom improvement [54,55]. It has been suggested that in some settings treatment may enhance perceived helplessness and result in decreased coping self-efficacy [56,57].

### Effect of social support on posttraumatic stress

Our finding that social support was inversely related to posttraumatic stress at T1 is consistent with previous findings [2,5]. However, social support was not related to symptom improvement from T1 to T2. Our finding indicates that the main positive effect of social support regard to posttraumatic stress is pronounced in the temporal proximity to the disaster. This is in line with previous findings where initial levels of social support did not predict the course of chronic PTSD symptoms [58]. The diminishing effect of social support in our study may be explained by persisting symptoms of avoidance and emotional numbing (Figure 2), which may diminish the supportive efforts of others, result in less social support or even deteriorate social relations [59].

### Emotional stability vs. neuroticism

In this study emotional stability proved consistently related to both low levels of posttraumatic stress at T1 and symptom improvement from T1 to T2. This could be expected as individuals with low levels of emotional stability (neuroticism) tend to respond emotionally negative to threat or loss [60]. The neuroticism subscale in Big Five Inventory (BFI) overlap to some extent with measures of posttraumatic stress (i.e getting easily stressed, irritated and having difficulties concentrating). Due to the study design it may be difficult to be sure of whether the measured personality traits are actual signs of neuroticism or merely represent symptoms of posttraumatic stress. However, instructions given for filling in IES-R scale largely differs from the instructions given for BFI. The first scale focuses on trauma-related symptoms experienced past seven days, while the latter scale queries whether a specific trait fits the person in general. In addition, a relation between neuroticism and posttraumatic stress has been reported in several other studies [11,61].

It was found that experience of other stressful life events was negatively related to symptom improvement (Table 2, *p* = 0.054). This finding may partly explain the strong inverse relation between neuroticism and symptom improvement in the present study as individuals with high neuroticism are likely to experience negative life events and tend to show an increased level of emotional reactivity to those events [62]. Consistent with previous studies our results imply that neuroticism plays an important role in the maintenance of long term posttraumatic stress symptoms [33,63]. Neuroticism is related to a variety of mental and physical disorders, partially mediated by inappropriate coping strategies [62,64].

### Methodological considerations

This study had some methodological advantages. Almost all Norwegians who were tourists in the disaster area were invited to participate, reducing sample selection bias. Both women and men in all age groups were represented in the study population. The participants were similar to the age- and gender-adjusted Norwegian population with regard to employment and marital status. Educational level, however was higher than average [22,65]. The relatively fast evacuation of the Norwegian tourists reduced the impact of secondary disaster stressors, limiting the impact of trauma to the primary tsunami experience. Finally, regardless of impairment level, our sample received affordable and easily accessible medical and psychiatric care as well as ample community support [52,66].

Limitations of our study include relatively low response rates, which imply interpreting the results with cautionous. Although 75% of T1 participants also participated at T2, our study population constituted only 27% of the total sample. Selective participation may have biased the results towards increased morbidity interfering with variables of interest like personality traits, coping behaviour and seeking treatment. Due to the directionality of the dropout bias, the included participants seem to represent most of the heavily exposed Norwegian tourists in the tsunami-stricken areas [22]. Thus the prevalence rates of cases based on high symptom score (IES-R total score ≥33) may be somewhat overestimated as compared to the whole adult population of Norwegian tourist who experienced the tsunami. However, the main focus of the current study was to investigate how variables were correlated to posttraumatic stress which is less likely to be affected by systematic response rate biases.

The information was gathered by the use of postal questionnaires. Thus, participants from the same household may have interacted during the filling-in process. This may have influenced the results [67].

Data regarding disaster exposure and peritraumatic reactions were ascertained retrospectively and may be affected by recall bias. Generally, the risk of recall bias increases as the time passes after the index traumatic event. A previous tsunami study showed that the memories for stressful events may amplify as time passes [22]. The study also showed that individuals with recall amplification did not improve in PTSD symptom severity from 6 to 24 months, whereas individuals who did not have recall amplification showed a reduction in PTSD symptoms over time. For this reason we have avoided the use of variables solely based on personal appraisal, and rather included more objectively defined criteria when measuring exposure.

Our results may not be applicable to other populations that were exposed to chronic stressors.

The fact that the participants were abroad on Christmas holiday suggests that they may represent a more privileged and healthy sub-population of the Norwegian population. However, we do not know in what way this could have affected the development and course of posttraumatic stress symptoms in the aftermath of the tsunami disaster.

## Conclusions

Approximately 70% of the Norwegian tourists in our study were directly exposed to the 2004 tsunami. A significant minority (20-30%) developed enduring posttraumatic stress symptoms in the aftermath. Our study demonstrates that in case of large scale emergencies, also where the survivors escape secondary disaster stressors, an initial assessment regarding disaster exposure and peritraumatic stress reactions seems crucial to predict psychiatric morbidity. Also, some knowledge about personality and previous psychosocial functioning may help to make a more accurate prediction regarding disaster survivors who are at increased risk for developing enduring posttraumatic stress symptoms. More detailed studies are needed to search for other variables to distinguish disaster victims who experience only the acute form from those who go on to develop the chronic form of PTSD. In particular, the effect of personality traits and stressful life events on the course of posttraumatic stress needs further investigation.

It proves that a significant number of disaster survivors continue to have enduring symptoms even when treatment is given. Thus continuous efforts are needed to obtain data on effectiveness of treatment in order to increase the use of effective treatments. Our findings imply that positive results obtained within research settings with regard to psychotherapeutic interventions for PTSD may not always have real-world effectiveness. Accordingly, the clinicians should be attentive to lack of effectiveness, and more importantly, any negative effects of ongoing treatment. There is need for more naturalistic studies and controlled studies with a less restricted inclusion criterion to expand the generalization of the findings.

## Endnotes

^a^A more detailed overview of the Tsunami research program and list of publications can be found at the NCVTS’ homepage: http://www.nkvts.no/fu/Sider/Tsunamien-2004-NKVTS-publikasjoner.aspx

## Competing interests

The authors declare that they have no competing interests.

## Authors’ contributions

AH developed the questionnaire and collected data at T2, performed the statistical analysis and drafted the manuscript. TH and LW were involved in developing the questionnaire and collecting data at T1. All authors participated in developing the hypothesis, interpretation of the data and revision of the manuscript. All authors read and approved the final manuscript.

## Authors’ information

All the authors have specialized in psychiatry and have docotoral degrees. LW has for several years been a professor of Disaster psychiatry at the University of Oslo. TH is a senior researcher at the Norwegian Centre for Violence and Traumatic Stress Studies and is professor of Disaster psychiatry at the University of Oslo. AH is currently working in an outpatient psychiatry clinic in Oslo, Akershus University Hospital.

## Pre-publication history

The pre-publication history for this paper can be accessed here:

http://www.biomedcentral.com/1471-244X/13/232/prepub
